# Molecular Sieve, Halloysite, Sepiolite and Expanded Clay as a Tool in Reducing the Content of Trace Elements in *Helianthus annuus* L. on Copper-Contaminated Soil

**DOI:** 10.3390/ma16051827

**Published:** 2023-02-23

**Authors:** Mirosław Wyszkowski, Jadwiga Wyszkowska, Natalia Kordala, Magdalena Zaborowska

**Affiliations:** 1 Department of Agricultural and Environmental Chemistry, University of Warmia and Mazury in Olsztyn, Łódzki 4 Sq., 10-727 Olsztyn, Poland; 2 Department of Soil Science and Microbiology, University of Warmia and Mazury in Olsztyn, Łódzki 3 Sq., 10-727 Olsztyn, Poland

**Keywords:** copper contamination, materials, *Helianthus annuus* L., trace elements

## Abstract

The aim of this study was to determine the effect of copper soil contamination on the trace element content of sunflower aerial parts and in roots. Another aim was to assess whether the introduction of selected neutralizing substances (molecular sieve, halloysite, sepiolite and expanded clay) into the soil could reduce the impact of copper on the chemical composition of sunflower plants. Copper soil contamination with 150 mg Cu^2+^ kg^−1^ of soil and 10 g of each adsorbent per kg of soil were used. Soil contamination with copper caused a significant increase in the content of this element in the aerial parts (by 37%) and roots (by 144%) of sunflower. Enriching the soil with the mineral substances reduced the amount of copper in the aerial parts of sunflower. Halloysite had the greatest effect (35%), while expanded clay had the smallest effect (10%). An opposite relationship was found in the roots of this plant. In copper-contaminated objects, a decrease in the content of cadmium and iron and an increase in the concentrations of nickel, lead and cobalt in the aerial parts and roots of sunflower were observed. The applied materials reduced the content of the remaining trace elements more strongly in the aerial organs than in the roots of sunflower. Molecular sieve had the greatest reducing effect on the content of trace elements in sunflower aerial organs, followed by sepiolite, while expanded clay had the least impact. The molecular sieve also reduced the content of iron, nickel, cadmium, chromium, zinc and, especially, manganese, whereas sepiolite reduced the content of zinc, iron, cobalt, manganese and chromium in sunflower aerial parts. Molecular sieve contributed to a slight increase in the content of cobalt, while sepiolite had the same effect on the content of nickel, lead and cadmium in the aerial parts of sunflower. All materials decreased the content of chromium in sunflower roots, molecular sieve—zinc, halloysite—manganese, and sepiolite—manganese and nickel. The materials used in the experiment, especially the molecular sieve and to a lesser extent sepiolite, can be used effectively to reduce the content of copper and some other trace elements, particularly in the aerial parts of sunflower.

## 1. Introduction

As a consequence of man’s agricultural and non-agricultural (industrial) activity, the properties and fertility of soil can worsen [1,2]. Particularly hazardous forms of soil degradation include those caused by the entry of foreign chemical substances to soil, both organic (e.g., crude oil chemicals, pesticides) [3] and inorganic ones (e.g., wastewater, municipal solid waste, deposited dust from the metallurgical industry) [2,4]. Trace elements accumulate in the environment, migrate within soil profiles, and are contained in various chemical compounds [2]. The development of industries and increasing mobility of human populations magnify the accumulation of trace elements in soil. There, they have persistent influence on both the soil dwelling organisms and the entire ecosystem because they do not undergo further transformations [5,6]. Soils used for food production that are contaminated with trace elements are therefore pose a direct threat to food safety (lower crop yields, poorer food/feed quality) [7,8] and to entire trophic chains [9]. Thus, it is necessary to implement sustainable measures aiming at the restoration of proper soil characteristics so that soils are able to perform their key economic and ecological functions [6].

Copper is an essential micronutrient in plants, where it plays an important role in such biological and physiological processes as photosynthesis, biosynthesis of proteins, transport of carbohydrates and mitochondrial respiration [10,11]. Copper is a co-factor of many enzymes (e.g., peroxidase dismutase, phenolic oxidase, plastocyanin) [12,13]; it also participates in conversions of nitrogen compounds and in plant tolerance mechanisms; in addition, it enhances the activity of nitrate reductase [12,14]. Copper also affects the permeability of cell membranes, metabolism of nucleic acids and the process of generative reproduction of plants (production of pollen and seeds) [15]. The copper content in plants typically ranges between 5 to 30 mg kg^−1^ [16]. Excess of this element has a negative effect on seed germination [17,18] and such metabolic process as respiration, enzyme activity [12,19] and photosynthesis (elevated photoinhibition, impaired structure of chloroplasts and composition of the membrane of thylakoids) [20]. Plants in which the physiological norms have been exceeded are observed to experience the following symptoms: distorted permeability of cell membranes [19], retarded growth, decreased mitotic activity of root cells, decreased size of conductive tissues [13,21] and chlorosis of leaves [22]. The toxic effect of copper, even towards tolerant plants, is observed when the soil content of copper exceeds 60–125 mg kg^−1^ [11]. Copper excess is also harmful to animals and people. It has been demonstrated that an excessively high concentration of copper can be cytotoxic, causing a number of health problems, e.g., stomachache, liver insufficiency [23], neurological disorders [24] and chronic anaemia [25].

Soil contamination with copper is a widespread problem in many countries [26], which dates back to the early days of industrial revolution [27]. The content of copper in soil continues to increase due to the application of copper-based fungicides and bactericides in horticulture and agriculture [16] as well as other pesticides [27]. Another source of metal-bearing contaminants is the disposal and recycling of waste, sewage sludge, copper-enriched animal manure in soil [27,28], as well as soil fertilisation and transport [26,29]. It has been demonstrated that in some areas occupied by special branches of agriculture, e.g., vineyards, the soil content of copper can exceed 500 mg kg^−1^ [18]. Excessive concentrations of copper are also detected in soils locally contaminated by mining or smelting (waste such as mine slag, slag from electric furnaces, solid waste from flue-gas desulfurization systems or floatation waste) and copper-bearing alloy processing [28,29,30].

There is ongoing search for remediation technologies applicable to contaminated soils, which would limit the negative impact of trace elements on a given biocenosis rather than remove such contaminants from the soil completely [29,31,32]. Attempts are made to achieve such an outcome through the immobilization of trace elements in soil by modifying the soil’s properties, especially reaction, content of organic substance and sorption properties [33,34], but also by growing certain plants or through the precipitation of trace elements as insoluble salts [35]. The amendment of soil for the purpose of its remediation with mineral materials, rich in aluminosilicates and clay minerals, or zeolites as contaminant adsorbents decreases amounts of phytoavailable forms of trace elements, leading to the restoration of homeostasis in the soil [36,37]. Neutralizing substances applied as soil amendments (including bentonite, molecular sieve, halloysite, sepiolite, biochar) bind harmful trace elements into insoluble compounds or organic-mineral complexes [38,39], which can remain immobilised in the soil for long periods under favourable conditions [40]. Moreover, these substances contribute to the enhanced microbiological and biochemical activity of soil, supplement the soil’s organic matter resources, and in consequence improve the soil’s condition and fertility [41,42,43]. Nanotechnology seems to have a special role in combination with bioremediation, which is currently the most promising and economical method of removing contaminants from the soil [32]. In situ stabilisation limits the plants uptake of trace elements from the soil [44,45,46] by reducing their solubility and bioavailability [39,47]. In addition, this technique is inexpensive, does not destroy the soil’s structure and biological activity [47], and prevents secondary contamination [48].

Effective, inexpensive and easily available soil amendments of natural origin are being sought, with view of using them broadly in in situ stabilisation of soils exposed to pressure of trace elements. Such materials include halloysite, sepiolite, molecular sieve and expanded clay. Sepiolite (Mg_4_Si_6_O_15_(OH)_2_ · 6H_2_O) is a natural hydrated magnesium silicate clay mineral with the structure similar to that of type 2:1 tri-octahedral silicates [49]. Some corner atoms on the surface of sepiolite sheets are bound to hydroxyl groups (Si-OH). These so-called silanol groups tend to be active sites accessible to ions or molecules, thereby playing the role of adsorption centres [50]. Molecular sieves are nano-porous materials, selective towards substances with specific molecules, which adsorb molecules with smaller dimensions [6]. The most common group of molecular sieves are zeolites, which are characterised by the crystal-like, uniform pore structure of [51]. They are also characterized by large specific surface, durability and the ability to regenerate [52]. Halloysite is a dioctahedral clay mineral, a 1:1 layer aluminosilicate [53]. It is characterized by high porosity (60–70%) and large specific area (56.2–58.0 m^2^ g^−1^), high ion-exchange capacity and ease of chemical and mechanical processing [54]. As a natural mineral with a nanotubular structure, halloysite has many technological applications, as a mineral adsorbent (it adsorbs toxic substances and trace elements from water and wastewater) [55] and as support for catalysts [56]. The cation-exchange capacity of halloysite varies from 2 to 60 cmol(+) kg^−1^ [57]. Expanded clay is produced from natural materials processed in rotary kilns heated up to 1100–1200 °C. Expanded clay particles are nearly round, with high total porosity (up to 80%) [58,59]. Moreover, it is non-flammable, chemically stable, non-absorbent (up to 20%) and resistant to mould, fungi or rodents [58,60]. Expanded clay is broadly used in environmental engineering, civil engineering, geotechnology and horticulture [61]. The application of nanomaterials improves soil properties and plays an important role in stimulating plant growth and shaping their proper chemical composition [51]. In view of the significant areas of soil contaminated with trace elements, it is therefore necessary to find materials with a high efficiency in sorbing or binding them in immobilised forms that would not be taken up in excessive quantities by plants.

Therefore, we hypothesised that (1) copper contamination of the soil would increase the content of this element and some other metals in the aerial parts and roots of sunflower, (2) the application of materials such as molecular sieve, halloysite, sepiolite and expanded clay to the soil would reduce the potential negative effects of copper contamination on the chemical composition of sunflower plants.

## 2. Material and Methods

### 2.1. Methodology of the Plant Growing Trials

The experiment was conducted on soil collected from the humic topsoil. It was a soil with a texture composition of sandy loam (60.63% of 0.0–2.0 mm fractions, 35.99% of 0.02–0.05 mm fractions and 3.38% of <0.002 mm fractions), classified as a Eutric Cambisol [62]. It was a controlled plant growing experiment, set up in polyethylene pots of 3.0 dm^3^ in capacity, conducted in the controlled environment of a greenhouse in Olsztyn (NE Poland). The basic properties of the soil used in the experiment were as follows: pH_KCl_—soil reaction in 1 mol KCl dm^−3^—6.00, hydrolytic acidity (HAC)—13.50 mmol (+) kg^−1^ DM, sum of exchangeable base cations (EBC)—145.00 mmol (+) kg^−1^ DM, cation exchange capacity (CEC)—158.50 mmol (+) kg^−1^ DM, base saturation (BS)—91.49%, content of: total nitrogen (N_tot_)—1.07 g kg^−1^ dry matter (DM), organic carbon (C_org_)—14.69 g kg^−1^ DM, phosphorus (P)—166.72 mg kg^−1^ DM, potassium (K)—171.31 mg kg^−1^ DM, magnesium (Mg)—443.21 mg kg^−1^ DM, copper (Cu)—4.20 mg kg^−1^ DM.

It was a typical, two-factorial experiment. The first factor was the copper soil contamination (without Cu—0 and with Cu—150 mg Cu^2+^ kg^−1^ of soil) used as CuSO_4_ · 5H_2_O, and the second factor was the application of different materials (adsorbents) used to reduce the negative effect of copper on plants. Molecular sieve, halloysite, sepiolite, and expanded clay were used, all at a dose of 10 g kg^−1^ DM of soil.

The product called Silosiv A3, which contains molecular sieve, was tested. It is a crystalline aluminium silicate with mircopores of the size 0.3 nm (Grace, Enriching Lives, Everywhere, USA). Halloysite (Al_2_Si_2_O_5_(OH)_4_) is a mineral of high ion exchange capacity owing to its large specific surface and porosity (diameter of pores between 10–20 nm), most often used as a mineral adsorbent (Halosorb, Intermark, Poland) [63]. Sepiolite (Mg_4_[Si_6_O_15_(OH)_2_]6H_2_O), or specifically the product named Sepiolite 60/100 (Sepiolsa Minersa Group, Spain) is hydrated magnesium silicate, with considerable adsorption properties, fibrous texture and the pore diameter of 8–1.6 mm [64,65]. Expanded clay is produced by baking loamy clay at a high temperature (1150–1200 °C). Same as molecular sieve, expanded clay is characterized by high porosity (GardenGURU, Poland) [66].

Additionally, in order to secure the nutritional requirements of the plants, each pot was supplied with fertilisers containing basic nutrients: nitrogen—110 mg N as CO(NH_2_)_2_, phosphorus—45 mg P as KH_2_PO_4_, potassium—110 mg K as KH_2_PO_4_ and KCl, and magnesium—20 mg as MgSO_4_ · 7H_2_O. Prior to mixing with copper, adsorbents and mineral fertilisers, soil was passed through a 5 mm mesh net sieve. Next (3 June), it was transferred to polyethylene pots and seeds of the plant were sown. Sunflower (*Helianthus annuus* L.) was chosen to be the test phytoremediation plant, as it is highly tolerant to soil contamination with heavy metals [67] and simultaneously plays an important role in the global food economy. In terms of the size of area cultivated for oilseed crops, the sunflower occupies the fourth place [68,69], and its biggest producers are Ukraine, Russia and Argentina [68]. This is due to the relatively low translocation of trace elements from the roots to the aerial parts of sunflower, despite the significant content of their soluble forms in the soil rizosphere. The translocation coefficient for sunflower may even be <1 [67]. Sunflower biomass can also be used as a renewable energy resource [70]. The pots were watered with distilled water, maintaining the soil moisture content at 60% of maximum water capacity throughout the entire plant growing period. The pot experiment was conducted in triplicate. During the harvest of sunflower plants at BBCH 35 stage (Biologische Bundesanstalt, Bundessortenamt and Chemical Scale)—43 days after sowing (14 July), plant samples were taken for laboratory analysis.

### 2.2. Physicochemical and Chemical Analyses of Soil and Plants

Before the experiment, the basic parameters of the soil used in the experiment were determined: the grain-size distribution using a Malvern Mastersizer 2000 Laser Diffraction (Malvern, Worcestershire, UK) [71], soil’s pH_KCl_ using a HI 2221 pH-meter (Hanna Instruments, Washington, UK) [72], hydrolytic acidity (HAC) and exchangeable base cations (EBC) using the Kappen method [73], total nitrogen content (N_tot_) using Buchi B-324 a distillation apparatus (Buchi, Flawil, Switzerland) [74], organic carbon (C_org_) on a Genesis 6 spectrophotometer (Thermo Electron Corporation, Switzerland, USA) [75], available phosphorus on a SQ118 spectrophotometer [76], potassium on a Jenway 6705 UV/VIS spectrophotometer (Jenway LTD, Staffordshire, UK) [76], magnesium on an atomic absorption spectrophotometer GBC 932AA (GBC Scientific Equipment, Braeside, Australia) [77], and copper by flame and electrothermal absorption spectrometry after extraction in aqua regia according to ISO 11047:1998 [78]. HAC and EBC values were used to calculate the soil’s cation exchange capacity (CEC) and base saturation (BS) [73].

Samples of aerial parts and roots of sunflower plants were cut, dried at a constant temperature of 60 °C and ground to a flour-like texture. Then, the plant material was digested in concentrated 65% nitric acid (HNO_3_ analytically pure, of the density of 1.40 g cm^−3^) according to US-EPA3051 [79] in a microwave oven MARS 6—CEM Corporation, Matthews, NC, USA. Concentrations of trace elements (copper, cadmium, lead, chromium, nickel, zinc, manganese, iron and cobalt) in digested plant samples were determined by atomic absorption spectrometry (ASA) using a SpectrAA 240FS spectrophotometer (Varian Inc., Mulgrave, VIC, Australia) [80]. While performing these analyses, reference materials by Fluka were used (Cu 38996, Cd 51994, Pb 16595, Cr 02733, Ni 42242, Zn 188227, Mn 63534, Fe 16596, Co 119785.0100), and the quality of the analyses was monitored using the certified reference material NCS ZC 73030 (Chinese National Analysis Center for Iron and Steel, Beijing, China).

### 2.3. Statistical Analysis

The research results were statistically processed in Statistica 13.3 [81], using a two-factorial analysis of variance and the Tukey’s test (HSD) to distinguish homogeneous groups at *p* = 0.01, and principal component analysis (PCA) and % of observed variation in order to determine the relationships between the content of trace elements in the aerial parts and roots of sunflower (dependent variables) versus the studied factors (soil contamination with copper and adsorbents—independent variables).

## 3. Results

**Figure 1:**
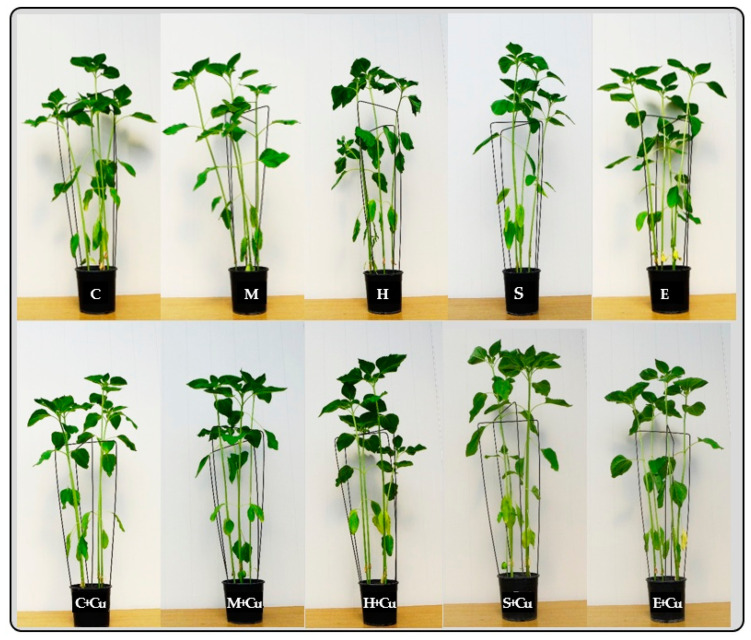
*Helianthus annuus* L. in the BBCH 34 phase. C—Control (uncontaminated soil); Cu—with Cu^2+^; M—molecular sieve; H—halloysite; S—sepiolite; E—expanded clay.

Plant growth and development and the content of trace elements in sunflower depended on both the contamination of soil with copper and the application of adsorbents (Table 1, Table 2 and Table 3, Figure 1). The materials used in copper-contaminated sites (mainly sepiolite and then halloysite) had a beneficial effect on plant growth and development (Figure 1). In non-copper-contaminated objects, their effect was relatively small.

The copper soil contamination (150 mg Cu^2+^ kg^−1^ of soil) caused a significant increase in the content of this element in aerial parts (by 37%) and in roots (by 144%) of sunflower grown in the control series, without the adsorbents application, relative to the uncontaminated pots (Table 1). The amendment of soil with the tested adsorbents had a positive effect by reducing the copper content in aerial parts of sunflower. Halloysite had the strongest effect while expanded clay produced the weakest influence, reducing the content of copper in sunflower’s aerial parts by 35% and 10%, respectively, in comparison to the control. Molecular sieve and sepiolite had a smaller but also reducing effect (13% and 20%, respectively) on the content of copper in aerial parts of the sunflower. Contrary effects were observed in the roots of this plant. Molecular sieve, sepiolite, halloysite and expanded clay caused an increase by 45%, 83%, 159% and 174%, respectively, in the copper content in sunflower’s roots relative to the pots where these substances were not added to soil. The increase in copper content in sunflower roots (as opposed to aerial parts) may be due to the positive effects of molecular sieve, sepiolite, halloysite and expanded clay on the physiological barrier, limiting excessive translocation of some elements from plant roots to aerial parts.

Aerial parts of sunflower plants grown in pots with copper-contaminated soil not enriched with any of the tested substances were found to have increased concentrations of nickel and manganese by 9%, zinc by 14%, lead by 23% and cobalt by 141%, while the content of cadmium, iron and chromium was lower by 15%, 18% and 19%, respectively, in comparison with uncontaminated objects (Table 1, Table 2 and Table 3). The materials added to soil had a much larger reducing effect on the content of trace elements in aerial parts than in roots of sunflower plants. The strongest reducing effect on the content of trace elements in sunflower’s aerial organs was produced by molecular sieve. In the series with copper-contaminated soil, molecular sieve reduced the content of iron by 5%, nickel by 8%, cadmium by 18%, chromium and zinc by 22% and manganese by as much as 44% in sunflower’s aerial parts relative to the object where no adsorbent was applied. The application of this material led to a slight increase, by 4%, in the content of cobalt in sunflower’s aerial parts. Halloysite, in turn, decreased the content of iron by 14%, cadmium by 18%, zinc by 23%, manganese by 26% and chromium by 43% while increasing the content of nickel by 5%, cobalt by 27% and lead by 123% in aerial parts of this plant grown in copper-contaminated soil. Sepiolite contributed to the reduction in the content of zinc by 12%, iron by 14%, cobalt by 18%, manganese by 40% and chromium by 52%, and to the elevation in the content of nickel by 31%, lead by 48% and cadmium by 100%. Expanded clay had the weakest positive and reducing effect on the content of trace elements in sunflower’s aerial organs. Expanded clay only decreased the content of chromium, by 44%, while causing an increase in the content of iron by 7%, nickel by 19%, lead by 23% and cadmium by 55% in aerial parts of sunflower plants harvested from the pots with copper-contaminated soil relative to those obtained from soil not enriched with this material.

In the series where no absorbents were applied, the copper soil contamination caused a decline in the content of cadmium and zinc by 57%, manganese by 59%, and iron by 76%, while leading to a higher content of nickel (by 17%), cobalt (by 68%), chromium (by as much as 156%) and lead (by 170%) in sunflower’s roots relative to the uncontaminated object (Table 1, Table 2 and Table 3). All the tested materials reduced the content of chromium in sunflower’s roots by 15% (molecular sieve) up to 33–36% (the other substances), while molecular sieve also reduced the root content of zinc by 4%, halloysite—the root content of manganese by 3%, and sepiolite—the root content of manganese and nickel by 3 and 9%, respectively, all relative to the objects without the application of these materials. In the remaining cases, the applied substances contributed to an increase or else had no effect on the trace elements content in sunflower’s roots.

**Figure 2:**
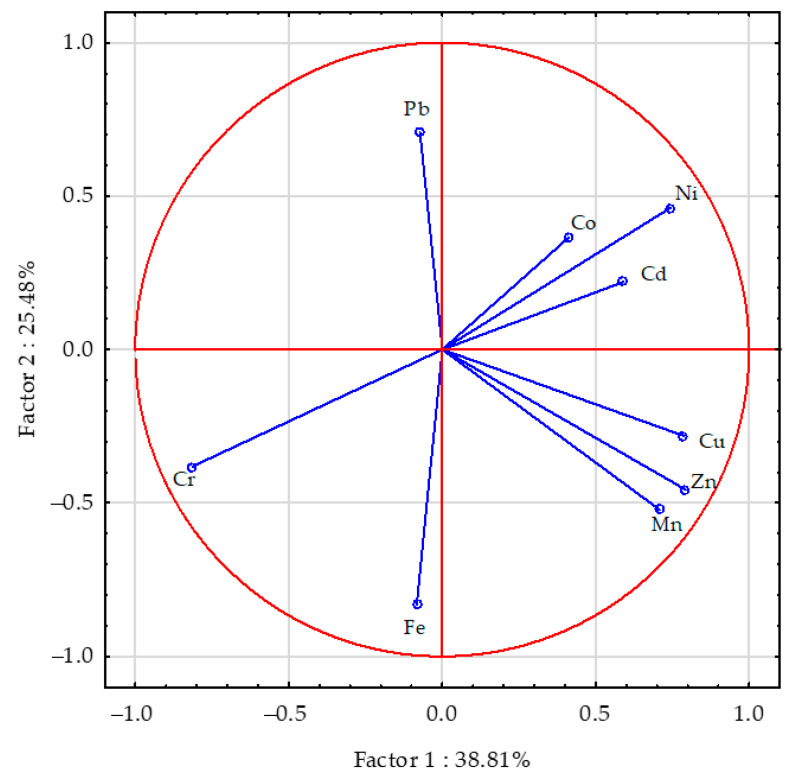
The trace elements content in the sunflower—*Helianthus annuus* L. aerial parts presented by a PCA analysis. Key: the vectors represent the analysed variables (content of Cu, Cd, Pb, Cr, Ni, Zn, Mn, Fe and Co).

**Figure 3:**
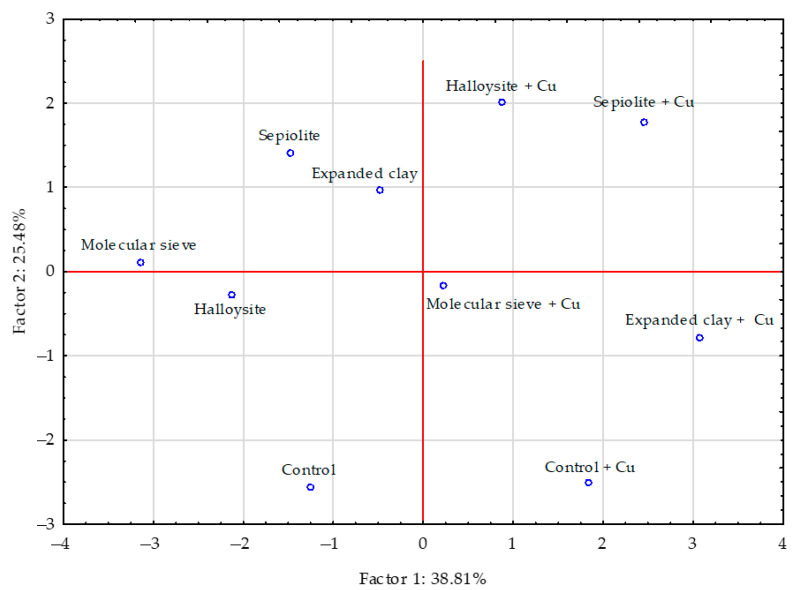
The influence of the materials on the trace elements content in the sunflower—*Helianthus annuus* L. aerial parts presented by a PCA analysis. Key: points indicate the samples with trace elements (uncontaminated: control—without materials, molecular sieve, halloysite, expanded clay; contaminated with copper: control + Cu —without materials + Cu, molecular sieve + Cu, halloysite + Cu, expanded clay + Cu).

The PCA performed showed that the contribution of all analysed factors accounted for 64.29% of the total correlation of the set of data (Figure 2). Vectors of the content of chromium, nickel, zinc, manganese and iron in sunflower aerial parts were similar in length, meaning that they produced comparable effects, whereas the vectors corresponding to the content of cobalt, cadmium and lead were shorter, indicating that their effect was weaker. The content of zinc in sunflower’s aerial parts was positively correlated with the content of manganese and copper, the content of nickel was positively correlated with cadmium and cobalt, that of copper with manganese, and cadmium with cobalt. Weaker positive correlations were determined between the content of chromium and iron, as well as nickel and lead in sunflower aerial parts. Negative correlations were detected between the content of chromium and nickel, and iron and lead, while weaker negative correlations occurred between cadmium and cobalt, and lead and manganese or zinc in a sunflower’s aerial parts. Sepiolite expanded clay and molecular sieve had a stronger effect on the content of trace elements in sunflower’s aerial parts than halloysite (Figure 3).

**Figure 4:**
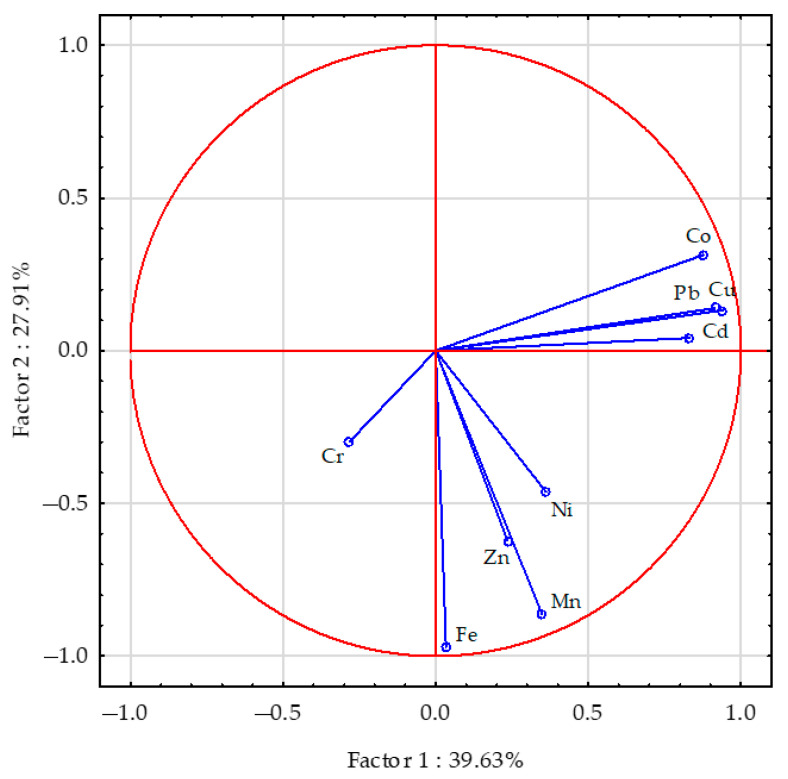
The trace elements content in the sunflower—*Helianthus annuus* L. roots presented by a PCA analysis. Key: the vectors represent the analysed variables (content of Cu, Cd, Pb, Cr, Ni, Zn, Mn, Fe and Co).

**Figure 5:**
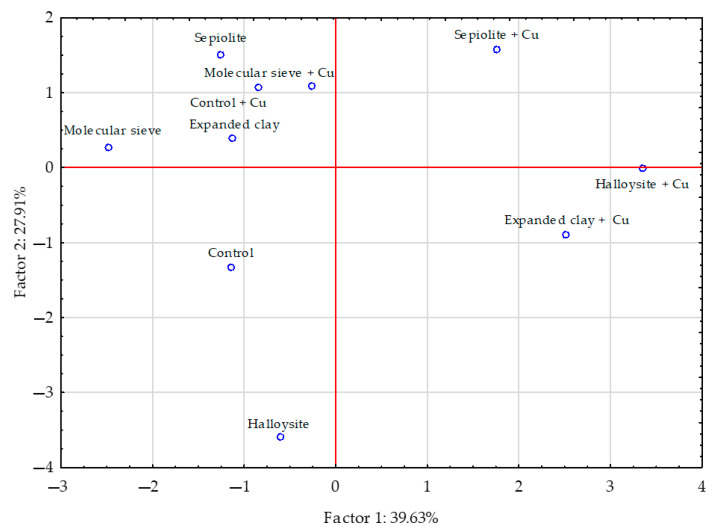
The influence of the materials on the trace elements content in the sunflower—*Helianthus annuus* L. roots presented by a PCA analysis. Key: points indicate the samples with trace elements (uncontaminated: control—without materials, molecular sieve, halloysite, expanded clay; contaminated with copper: control + Cu—without materials, molecular sieve + Cu, halloysite + Cu, expanded clay + Cu).

The aggregated share of the analysed factors in the total correlation of the data sets was 67.54% (Figure 4). Quite large variation was demonstrated in the length of vectors representing the content of particular trace elements in sunflower roots. The zinc, nickel and especially chromium vectors were much shorter than those of the other elements, indicating that these three elements were less important. The content of copper and lead in sunflower’s roots was positively correlated with cobalt and cadmium, the root content of cobalt was positively correlated with cadmium, that of manganese with iron and, less strongly, with zinc and nickel, and the root content of iron was correlated positively with zinc and nickel. Weak negative correlations were also determined between the content of chromium and iron versus concentrations of cobalt, copper, lead and cadmium in sunflower’s roots. The scattering of points in Figure 5 suggests that the content of trace elements in the roots of sunflower grown in copper-contaminated soil was most strongly influenced by sepiolite and molecular sieve.

**Figure 6:**
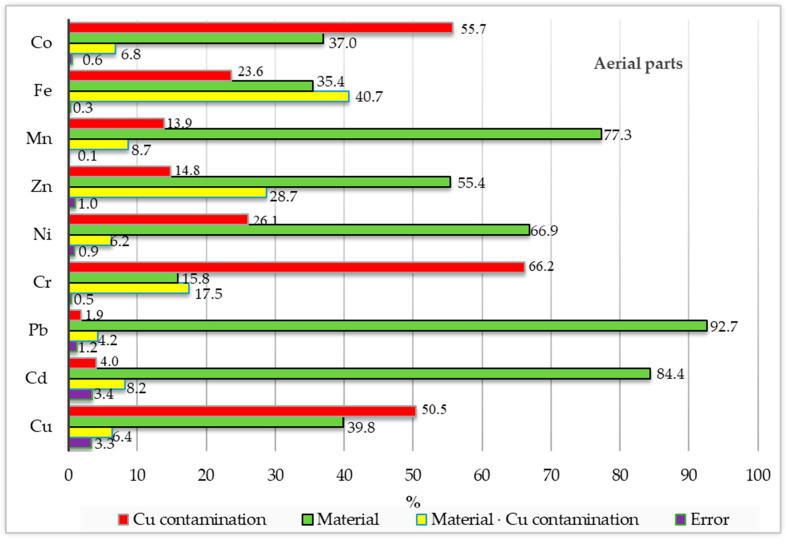

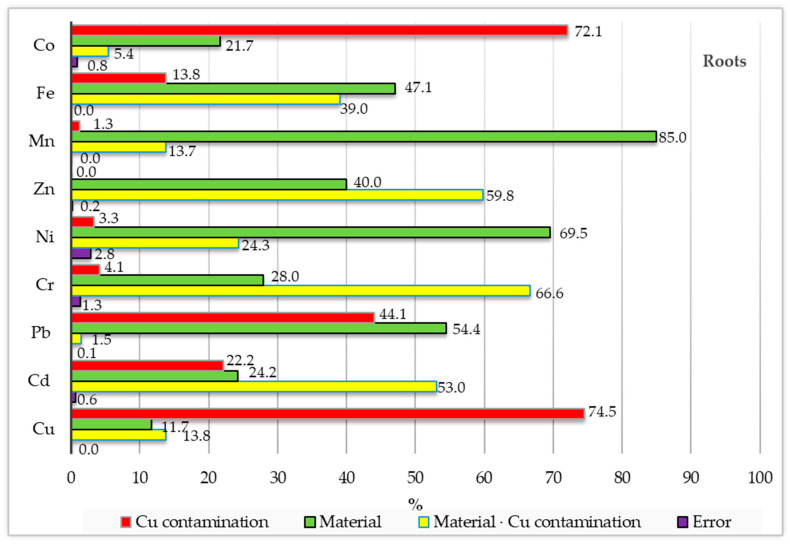
Relatively influence of factors on trace elements contents in the sunflower—*Helianthus annuus* L. (in percent).

Figure 6 illustrates the percentage of observed variation, showing the cumulative impact of the analysed factors (soil contamination with copper and the application of different materials into soil) on the content of trace elements in sunflower’s aerial organs and roots. Soil contamination with copper had a stronger effect only on the content of copper (50.5%), chromium (66.2%) and cobalt (55,7%) in sunflower’s aerial parts, and on the content of copper (74.5%) and cobalt (72.1%) in sunflower’s roots than the applied adsorbents did (39.8%, 15.8% and 37.0% in aerial parts and 11.7% and 21.7% in roots, respectively). The adsorbents applied to soil had a stronger impact than copper on the content of lead (92.7%), cadmium (84.4%), manganese (77.3%), nickel (66.9%), zinc (55.4%) and iron (35.4%) in aerial parts, and on the concentration of manganese (85.0%), nickel (69.5%), lead (54.4%), iron (47.1%), zinc (40.0%), chromium (28.0%) and cadmium (24.2%) in roots of sunflower plants. The soil contamination with copper had a considerable effect on the content of nickel (26.1%) and iron (23.6%) in aerial parts, and on the concentration of lead (44.1%) and cadmium (22.2%) in roots. It is worth emphasizing that the applied materials in the interaction with the soil contamination with copper had the strongest effect on the iron content in the aerial parts and on the concentrations of chromium, zinc and cadmium in roots of sunflower.

## 4. Discussion

In this study, the contamination of soil with copper in a dose of 150 mg kg^−1^ was conducive to the accumulation of most trace elements in both aerial parts and roots of sunflower. The biggest changes were recorded for copper, whose content increased 1.4-fold in aerial biomass and 2.4-fold in roots of the test plant. A similar tendency was observed in their experiment by Wyszkowski and Brodowska [82]. These authors demonstrated that the soil contamination with copper in an amount of 200 mg kg^−1^ led to a 27-fold increase in the content of this element in aerial parts of maize as well as the greater accumulation of cobalt (by 75%), manganese (by 49%) and iron (by 30%) relative to the control. Copper may have had an antagonistic effect of on the translocation of iron and zinc from the stem to leaves of plants, e.g., rapeseed [83]. In the above experiment, the lowest applied dose of copper (50 mg Cu kg^−1^) favoured the accumulation of manganese, iron and zinc increasing their content by 13%, 5% and 8%, respectively, compared to the control. This relationship was not observed in this experiment. These differences were most probably due to the different levels of copper contamination (here 150 mg kg^−1^ of soil) and the different plant species tested.

Copper contamination of soil influences soil physical properties, thereby modifying the translocation of trace elements in the soil–plant system [26]. As reported by Żołnowski and Wyszkowski [40], the copper contamination of soil resulted in a decreased soil pH and increased hydrolytic acidity (HAC). It also led to a decrease in total exchangeable bases (TEB), which resulted in the decreased cation exchange capacity (CEC) and base saturation (BS). Similar observations were reported by Guo et al. [84]. The decreased pH of soil exposed to copper pressure is most probably due to the replacement of H^+^ by Cu^2+^ ions, and desorption of H^+^ ions to the soil solution [26]. A decrease in the soil reaction to slightly acidic or acidic increased the solubility of chemical bonds of trace elements, thereby raising their mobility and content of phytoavailable forms [85]. In these conditions, trace elements migrate to the soil solution and become potentially available to plants. This may explain the higher content of copper, nickel, zinc, manganese and cobalt in the aerial parts and that of copper, lead, chromium and cobalt in the roots of sunflower found in this experiment.

Copper, same as other trace elements, does not undergo degradation [86], and due to the poor ability of soil for self-purification, copper can accumulate in the topsoil for many years, posing a local threat to groundwater, plants and animals [23]. As the actual uptake of trace elements by crops depends on the phytoavailability rather than concentration of these elements [49], chemical stabilisation of contaminants is an economically viable and environmentally reasonable solution. As reported by Wyszkowski and Brodowska [82], mineral amendments such as bentonite and zeolite change the soil pH and immobilize trace elements more effectively than organic soil amendments.

Nanomaterials application to soil plays an important role in limiting the contaminants uptake by plants [51]. In our experiment, all of the soil amendments tested, exception of expanded clay, were effective in reducing the negative effects of soil contamination with copper. The adsorbents introduced to soil decreased the bioavailability of copper, chromium, zinc, manganese and iron, limiting their translocation to aerial parts of sunflower. Sepiolite and molecular sieve proved to be most effective in the restoration of soil’s homeostasis. The application of zeolite to soil has a beneficial effect on soils with low pH by raising their reaction [87,88], hence making it possible to immobilize contaminants effectively. As the soil pH raises, the solubility of chemical bonds of trace elements decreases while their adsorption on soil colloids increases, which leads to a decline in amounts of mobile and bioavailable forms of these elements [88]. High effectiveness of molecular sieves in the immobilization of cadmium and lead in contaminated soil has been confirmed by Huang-Ping and Shu-Hao [89]. Farooqi et al. [90] also demonstrated the usefulness of zeolite for the in situ stabilisation of agricultural soil fertilised for many years with wastewater. When this mineral material was applied to soil in an amount of 2% (*w*/*w*), the researchers noted reduced accumulation of copper (by 43%), cadmium (by 97%), nickel (by 70%) and lead (by 100%) in fruits of Solanum melongena. Under the same conditions, the content of copper, cadmium, nickel and lead in soil decreased by 55%, 47%, 55% and 58%, respectively, in comparison with the control series (without zeolite). Similar conclusions were reported by Chen et al. [91], who observed a decreased content of cadmium and lead in wheat following the application of zeolite to soil.

In our experiment, sepiolite statistically significantly reduced the content of copper, chromium, zinc, manganese, iron and cobalt in the aerial biomass and chromium in the root of sunflower. This effect can be attributed to the large specific surface area of sepiolite (230–320 m^2^ g^−1^) [92], cation exchange capacity and soil pH regulation [49,93]. This is confirmed by other authors [94,95,96]. Liang et al. [95] proved that combined application of sepiolite and phosphorus fertiliser resulted in a decrease in the content of cadmium and lead in *Lactuca sativa* L. by 52% and 55%, respectively, relative to the control. The usefulness of sepiolite for in situ stabilisation of soil polluted with contaminants from the mining industry was also demonstrated by Lin et al. [94]. These researchers used a 4% addition of this mineral product, which enabled the reduction in the water-soluble fractions of cadmium and zinc by 57% and 41%, and their extractable fractions by 43% and 25%, respectively. The mobility of elements in the soil analysed also decreased by 69% (for cadmium) and by 52% (for zinc). The reduced translocation of cadmium and lead from soil to roots and shoots of spinach following the soil application of sepiolite was also shown by Sun et al. [97]. When this material was added to contaminated soil in a dose of 5%, the content of cadmium and lead in spinach roots decreased by 51% and 46%, respectively, while their concentration in spinach shoots was lower by 46% and 66%, respectively, in comparison with the control (without any addition of the neutralizing substance). These authors also determined that increasing doses of sepiolite resulted in the soil pH rising from 7.72 to 8.03.

An important property of the clay materials applied in our experiment is their ability to interact with inorganic substances. As a result of such interactions, the soil-borne, bioavailable forms of trace elements can transform into less readily available forms. This occurs through the adsorption on the surface of mineral grains, cation exchange or intercalation [98]. Once a mineral supplement is introduced to the contaminated soil, certain isomorphic substitutions occur in the tetrahedral network layers of the mineral, which leads to the formation of negatively charged adsorption sites. Such sites are then occupied by exchangeable cations, which compensate negative charges [93,99] and become immobilized. Soil amendments in the form of minerals, by inducing the immobilization of trace elements, can be therefore useful for the remediation of copper contaminated soils.

From the discussion of the research results obtained, it is difficult to find research on this topic with sunflower and many other plant species. The promising results obtained with these sorbents indicate that there is a need to continue research on the topic addressed in this paper. Possible future research should focus on plant species other than sunflower. These should mainly be energy crops whose biomass can be used for energy production. It would be advisable to carry out research under environmental conditions on a larger scale and with more trace elements. It would also be interesting to perform studies on the possibility of neutralising heavy metals in various interactions.

## 5. Conclusions

Soil contamination with copper caused a significant increase in the content of this element in the aerial parts (by 37%) and roots (by 144%) of sunflower. The enrichment of soil with the mineral substances reduced the amount of copper in the aerial parts of sunflower. The strongest 35% effect was achieved by halloysite, while the weakest 10% was obtained with expanded clay. Reverse relationships were found in the roots of this crop. In copper-contaminated objects in the series without adsorbents, a decrease in the content of cadmium and iron and an increase in the concentrations of nickel, lead and cobalt in aerial parts and roots of sunflower were observed. Concerning the content of the other analysed elements, either a reverse direction of change or no significant change was observed in both aerial parts and roots of sunflower. The applied materials reduced the content of the remaining trace elements more strongly in the aerial organs than in the roots of sunflowers. The molecular sieve had the highest reducing effect on the content of trace elements in sunflower aerial parts, followed by sepiolite, while expanded clay had the least effect. The molecular sieve also reduced the content of iron, nickel, cadmium, chromium, zinc and, especially, manganese, whereas sepiolite reduced the content of zinc, iron, cobalt, manganese and chromium in the aerial parts of the sunflower. The molecular sieve contributed to a slight increase in the content of cobalt, while sepiolite had the same effect on the content of nickel, lead and cadmium in aerial parts of sunflower. All materials decreased the content of chromium in the roots of the sunflower, molecular sieve—zinc, halloysite—manganese, and sepiolite—manganese and nickel. The tested materials either contributed to an increase or had no effect on the content of the other trace elements in sunflower roots.

## Figures and Tables

**Table 1:** Content of copper, cadmium and lead in sunflower—*Helianthus annuus* L. (mg kg^−1^ DM).

Object	Aerial Parts			Roots		
	Without Cu	With Cu	% Change with Cu/without Cu	Without Cu	With Cu	% Change with Cu/without Cu
Copper content (mg kg^−1^ DM)						
Control	1.628 ± 0.017 ^bc^	2.234 ± 0.124 ^e^	37.2	4.388 ± 0.016 ^c^	10.720 ± 0.044 ^d^	144.3
Molecular sieve	1.486 ± 0.028 ^ab^	1.936 ± 0.116 ^d^	30.3	2.484 ± 0.024 ^a^	15.570 ± 0.078 ^e^	526.8
Halloysite	1.282 ± 0.010 ^a^	1.449 ± 0.028 ^ab^	13.0	3.611 ± 0.045 ^b^	27.737 ± 0.026 ^g^	668.1
Sepiolite	1.430 ± 0.003 ^ab^	1.792 ± 0.025 ^cd^	25.3	3.533 ± 0.070 ^b^	19.576 ± 0.062 ^f^	454.1
Expanded clay	1.501 ± 0.043 ^ab^	2.012 ± 0.076 ^de^	34.0	2.651 ± 0.074 ^a^	29.419 ± 0.041 ^h^	1009.7
Average	1.465 ^A^	1.885 ^B^	28.6	3.333 ^A^	20.604 ^B^	518.1
Cadmium content (mg kg^−1^ DM)						
Control	0.013 ± 0.002 ^cd^	0.011 ± 0.001 ^bc^	−15.4	0.014 ± 0.001 ^b^	0.006 ± 0.001 ^a^	−57.1
Molecular sieve	0.009 ± 0.001 ^ab^	0.009 ± 0.001 ^ab^	0.0	0.007 ± 0.001 ^a^	0.007 ± 0.001 ^a^	0.0
Halloysite	0.006 ± 0.001 ^a^	0.009 ± 0.001 ^ab^	50.0	0.005 ± 0.001 ^a^	0.032 ± 0.001 ^d^	540.0
Sepiolite	0.016 ± 0.001 ^d^	0.022 ± 0.001 ^e^	37.5	0.006 ± 0.001 ^a^	0.018 ± 0.001 ^c^	200.0
Expanded clay	0.014 ± 0.001 ^cd^	0.017 ± 0.001 ^d^	21.4	0.012 ± 0.001 ^b^	0.017 ± 0.001 ^c^	41.7
Average	0.012 ^A^	0.014 ^B^	17.2	0.009 ^A^	0.016 ^B^	81.8
Lead content (mg kg^−1^ DM)						
Control	0.174 ± 0.005 ^a^	0.214 ± 0.030 ^a^	23.0	0.128 ± 0.006 ^b^	0.346 ± 0.001 ^c^	170.3
Molecular sieve	0.197 ± 0.002 ^a^	0.211 ± 0.018 ^a^	7.1	0.099 ± 0.011 ^a^	0.351 ± 0.002 ^c^	254.5
Halloysite	0.388 ± 0.011 ^d^	0.477 ± 0.007 ^e^	22.9	0.408 ± 0.002 ^d^	0.749 ± 0.003 ^g^	83.6
Sepiolite	0.337 ± 0.009 ^c^	0.316 ± 0.004 ^c^	−6.2	0.439 ± 0.008 ^e^	0.765 ± 0.010 ^g^	74.3
Expanded clay	0.261 ± 0.001 ^b^	0.264 ± 0.005 ^b^	1.1	0.337 ± 0.008 ^c^	0.697 ± 0.010 ^f^	106.8
Average	0.271 ^A^	0.296 ^B^	9.2	0.282 ^A^	0.582 ^B^	106.1

Values are average ± standard deviation. Homogeneous groups indicated by different letters (^A,B^ for Cu contamination, ^a–h^ for interaction between Cu contamination and substance type) were calculated separately for each trace element. There are significant differences at *p* ≤ 0.01 (Anova, Tukey’s HSD test).

**Table 2:** Content of chromium, nickel and zinc in sunflower—*Helianthus annuus* L. (mg kg^−1^ DM).

Object	Aerial Parts			Roots		
	Without Cu	With Cu	% Change with Cu/without Cu	Without Cu	With Cu	% Change with Cu/without Cu
Chromium content (mg kg^−1^ DM)						
Control	2.271 ± 0.090 ^d^	1.844 ± 0.023 ^c^	−18.8	1.825 ± 0.069 ^a^	4.670 ± 0.081 ^d^	155.9
Molecular sieve	3.491 ± 0.052 ^g^	1.435 ± 0.012 ^b^	−58.9	3.913 ± 0.070 ^c^	3.992 ± 0.014 ^cd^	2.0
Halloysite	2.799 ± 0.081 ^e^	1.049 ± 0.001 ^a^	−62.5	6.187 ± 0.064 ^e^	2.996 ± 0.074 ^b^	−51.6
Sepiolite	3.118 ± 0.069 ^f^	0.877 ± 0.038 ^a^	−71.9	2.503 ± 0.056 ^ab^	3.052 ± 0.028 ^b^	21.9
Expanded clay	1.715 ± 0.040 ^c^	1.027 ± 0.056 ^a^	−40.1	6.292 ± 0.023 ^e^	3.137 ± 0.090 ^b^	−50.1
Average	2.679 ^B^	1.246 ^A^	−53.5	4.144 ^B^	3.569 ^A^	−13.9
Nickel content (mg kg^−1^ DM)						
Control	2.877 ± 0.041 ^b^	3.130 ± 0.024 ^c^	8.8	2.600 ± 0.063 ^ab^	3.035 ± 0.081 ^b^	16.7
Molecular sieve	2.485 ± 0.042 ^a^	2.878 ± 0.045 ^b^	15.8	2.377 ± 0.028 ^a^	2.998 ± 0.080 ^b^	26.1
Halloysite	2.343 ± 0.025 ^a^	3.290 ± 0.077 ^cd^	40.4	3.729 ± 0.054 ^c^	2.821 ± 0.070 ^ab^	−24.3
Sepiolite	3.415 ± 0.020 ^d^	4.102 ± 0.078 ^f^	20.1	2.610 ± 0.077 ^ab^	2.752 ± 0.049 ^ab^	5.4
Expanded clay	3.380 ± 0.041 ^d^	3.727 ± 0.076 ^e^	10.3	3.618 ± 0.088 ^c^	4.463 ± 0.082 ^d^	23.4
Average	2.900 ^A^	3.425 ^B^	18.1	2.987 ^A^	3.214 ^B^	7.6
Zinc content (mg kg^−1^ DM)						
Control	28.32 ± 0.26 ^d^	32.25 ± 0.14 ^e^	13.9	110.77 ± 1.96 ^g^	48.09 ± 0.31 ^c^	−56.6
Molecular sieve	23.18 ± 0.05 ^a^	25.11 ± 0.36 ^bc^	8.3	38.31 ± 1.67 ^b^	46.18 ± 1.04 ^c^	20.5
Halloysite	28.22 ± 0.08 ^d^	24.80 ± 0.10 ^b^	−12.1	71.40 ± 0.05 ^f^	61.51 ± 0.10 ^e^	−13.9
Sepiolite	25.64 ± 0.30 ^bc^	28.53 ± 0.22 ^d^	11.3	29.18 ± 0.18 ^a^	56.50 ± 0.05 ^d^	93.6
Expanded clay	26.38 ± 0.06 ^c^	32.25 ± 0.28 ^e^	22.3	33.41 ± 0.47 ^a^	70.30 ± 0.74 ^f^	110.4
Average	26.35 ^A^	28.59 ^B^	8.5	56.61 ^A^	56.52 ^A^	−0.2

Values are average ± standard deviation. Homogeneous groups indicated by different letters (^A,B^ for Cu contamination, ^a–g^ for interaction between Cu contamination and substance type) were calculated separately for each trace element. There are significant differences at *p* ≤ 0.01 (Anova, Tukey’s HSD test).

**Table 3:** Content of manganese, iron and cobalt in sunflower—*Helianthus annuus* L. (mg kg^−1^ DM).

Object	Aerial Parts			Roots		
	Without Cu	With Cu	% Change with Cu/without Cu	Without Cu	With Cu	% Change with Cu/without Cu
Manganese content (mg kg^−1^ DM)						
Control	29.49 ± 0.14 ^f^	32.11 ± 0.43 ^g^	8.9	55.19 ± 0.61 ^d^	22.61 ± 0.07 ^a^	−59.0
Molecular sieve	15.74 ± 0.06 ^a^	18.06 ± 0.09 ^b^	14.7	33.79 ± 0.19 ^c^	22.95 ± 0.73 ^a^	−32.1
Halloysite	20.76 ± 0.09 ^d^	23.86 ± 0.10 ^e^	14.9	134.68 ± 0.77 ^g^	99.66 ± 0.67 ^f^	−26.0
Sepiolite	16.37 ± 0.26 ^a^	19.29 ± 0.10 ^c^	17.8	25.88 ± 0.07 ^b^	21.85 ± 0.18 ^a^	−15.6
Expanded clay	21.22 ± 0.04 ^d^	32.85 ± 0.07 ^g^	54.8	26.52 ± 0.08 ^b^	67.41 ± 0.63 ^e^	154.2
Average	20.72 ^A^	25.23 ^B^	21.8	55.21 ^B^	46.90 ^A^	−15.1
Iron content (mg kg^−1^ DM)						
Control	24.94 ± 0.01 ^f^	20.34 ± 0.37 ^c^	−18.4	1911.71 ± 4.43 ^g^	458.62 ± 7.31 ^a^	−76.0
Molecular sieve	18.84 ± 0.09 ^b^	19.31 ± 0.20 ^b^	2.5	1444.87 ± 7.60 ^e^	810.06 ± 7.41 ^cd^	−43.9
Halloysite	23.50 ± 0.16 ^e^	17.41 ± 0.02 ^a^	−25.9	3195.57 ± 6.25 ^h^	1437.98 ± 2.69 ^e^	−55.0
Sepiolite	20.20 ± 0.04 ^c^	17.48 ± 0.07 ^a^	−13.5	672.19 ± 4.03 ^b^	752.27 ± 6.58 ^c^	11.9
Expanded clay	20.06 ± 0.05 ^c^	21.76 ± 0.01 ^d^	8.5	827.13 ± 8.16 ^d^	1704.58 ± 10.39 ^f^	106.1
Average	21.51 ^B^	19.26 ^A^	−10.5	1610.29 ^B^	1032.70 ^A^	−35.9
Cobalt content (mg kg^−1^ DM)						
Control	0.037 ± 0.001 ^a^	0.089 ± 0.004 ^e^	140.5	0.111 ± 0.009 ^a^	0.186 ± 0.002 ^c^	67.6
Molecular sieve	0.040 ± 0.001 ^ab^	0.093 ± 0.002 ^e^	132.5	0.104 ± 0.001 ^a^	0.215 ± 0.0052 ^d^	106.7
Halloysite	0.093 ± 0.002 ^e^	0.113 ± 0.002 ^f^	21.5	0.155 ± 0.003 ^b^	0.264 ± 0.012 ^e^	70.3
Sepiolite	0.046 ± 0.004 ^bc^	0.073 ± 0.002 ^d^	58.7	0.154 ± 0.002 ^b^	0.256 ± 0.007 ^e^	66.2
Expanded clay	0.050 ± 0.001 ^c^	0.090 ± 0.003 ^e^	80.0	0.168 ± 0.005 ^bc^	0.216 ± 0.003 ^d^	28.6
Average	0.053 ^A^	0.092 ^B^	72.2	0.138 ^A^	0.227 ^B^	64.3

Values are average ± standard deviation. Homogeneous groups indicated by different letters (^A,B^ for Cu contamination, ^a–h^ for interaction between Cu contamination and substance type) were calculated separately for each trace element. There are significant differences at *p* ≤ 0.01 (Anova, Tukey’s HSD test).

## Data Availability

All data are available in the manuscripts and from the authors.
